# The Role of α_7_ Nicotinic Acetylcholine Receptor in Modulation of Heart Rate Dynamics in Endotoxemic Rats

**DOI:** 10.1371/journal.pone.0082251

**Published:** 2013-12-10

**Authors:** Roham Mazloom, Golnar Eftekhari, Maryam Rahimi, Vahid Khori, Sohrab Hajizadeh, Ahmad R. Dehpour, Ali R. Mani

**Affiliations:** 1 Department of Physiology, School of Medical Sciences, Tarbiat Modares University, Tehran, Iran; 2 Department of Pharmacology, School of Medicine, Tehran University of Medical Sciences, Tehran, Iran; 3 Ischemic Disorders Research Center, University of Medical Sciences, Gorgan, Iran; University of Cincinnati, United States of America

## Abstract

Previous reports have indicated that artificial stimulation of the vagus nerve reduces systemic inflammation in experimental models of sepsis. This phenomenon is a part of a broader cholinergic anti-inflammatory pathway which activates the vagus nerve to modulate inflammation through activation of alpha7 nicotinic acetylcholine receptors (α7nACHR). Heart rate variability represents the complex interplay between autonomic nervous system and cardiac pacemaker cells. Reduced heart rate variability and increased cardiac cycle regularity is a hallmark of clinical conditions that are associated with systemic inflammation (e.g. endotoxemia and sepsis). The present study was aimed to assess the role of α7nACHR in modulation of heart rate dynamics during systemic inflammation. Systemic inflammation was induced by injection of endotoxin (lipopolysaccharide) in rats. Electrocardiogram and body temperature were recorded in conscious animals using a telemetric system. Linear and non-linear indices of heart rate variability (e.g. sample entropy and fractal-like temporal structure) were assessed. RT-PCR and immunohistochemistry studies showed that α7nACHR is expressed in rat atrium and is mainly localized at the endothelial layer. Systemic administration of an α7nACHR antagonist (methyllycaconitine) did not show a significant effect on body temperature or heart rate dynamics in naïve rats. However, α7nACHR blockade could further reduce heart rate variability and elicit a febrile response in endotoxemic rats. Pre-treatment of endotoxemic animals with an α7nACHR agonist (PHA-543613) was unable to modulate heart rate dynamics in endotoxemic rats but could prevent the effect of endotoxin on body temperature within 24 h experiment. Neither methyllycaconitine nor PHA-543613 could affect cardiac beating variability of isolated perfused hearts taken from control or endotoxemic rats. Based on our observations we suggest a tonic role for nicotinic acetylcholine receptors in modulation of heart rate dynamics during systemic inflammation.

## Introduction

Cardiac rhythm displays a complex dynamics in physiological state which is due to non-linear interaction between cardiac pacemaker cells and the autonomic nervous system [1]. A variety of methods have been developed to assess cardiac cycle dynamics in health and disease [2]. These methods have provided evidence to show that heart rate dynamics is altered during systemic inflammation (e.g. in patients with sepsis) [2]. Therefore, heart rate variability (HRV) analysis has been used for non-invasive monitoring of patients with sepsis [3]. These reports have shown that decreased HRV and increased cardiac cycle regularity has diagnostic and prognostic value in patients with systemic inflammatory response syndrome [3–5]. Experimental studies in animal models have also indicated that endotoxemia is associated with a significant reduction in HRV [6–9]. Likewise, systemic administration of interleukin-6 (IL-6) decreases HRV in mice [10]. The mechanism of increased regularity of cardiac cycle during systemic inflammation in not very well understood. Recent studies have shown that partial uncoupling of cardiac pacemaker cells from cholinergic neural control may play a role in endotoxin-induced loss of HRV in experimental models [9,11]. Moreover, incubation of mouse isolated atria with recombinant IL-6 was associated with a significant reduction in chronotropic response to carbacholine (a cholinergic agonist) *in vitro* [10]. These reports indicate that activation of inflammatory pathways in cardiac pacemaker cells might affect its responsiveness to parasympathetic autonomic nervous control.

Recent investigations suggest that activation of vagus nerve can suppress pro-inflammatory cytokine levels in liver and spleen in mice [12]. Furthermore, electrical stimulation of the vagus nerve inhibits tumor necrosis factor-alpha (TNF-α) synthesis in wild-type mice, but fails to inhibit TNF-α synthesis in alpha7-nicotinic acetylcholine receptor (α7nACHR) deficient mice [13]. Since this discovery, the contribution of α7nACHR in modulation of inflammation has been shown in a variety of model systems [14]. α7nACHR is a homopentameric ligand-gated ion channel [14] which acts as a calcium channel [15] as well as a ligand-dependent coupler to secondary and tertiary messenger systems [16]. α7nACHR is distributed in several tissues [17], especially in macrophages which are important producer of inflammatory cytokines [14]. Atrial cells receive dense cholinergic innervation and express muscarinic cholinergic receptors [18]. Little is known about the expression of α7nACHR in atrial cells; however previous studies in rats have shown that cardiomyocytes exhibit immunoreactivity for α7nACHR at all embryonic developmental stage, although adult cardiomyocytes immunoreactivity for this receptor is weak [19]. The role of α7nACHR in modulation of cardiac function is not well understood. Deck et al. reported that α7nACHR is not required for parasympathetic control of heart rate in mouse [20]. However the role of this receptor in modulation of inflammatory process in the atria has not been investigated.

Sepsis and endotoxemia are associated with decreased HRV and impaired atrial chronotropic responsiveness to muscarinic cholinergic neural control [9]. The underlying mechanism of this phenomenon is uncertain, and the role of anti-inflammatory mediators in modulation of heart rate dynamics during systemic inflammation is not well understood. Fairchild et al. demonstrated that dexamethasone shortened but did not eliminate LPS-induced HRV depression in mice [8]. Likewise, Alvarez et al. reported that administration of a glucocorticoid before endotoxin reduced cytokine levels but did not affect HRV indices in human volunteers [21]. α7nACHR as a key component of cholinergic anti-inflammatory pathway have been shown to modulate local and systemic inflammation in a variety of model systems. However the role of this receptor in sepsis-induced HRV depression is not clear. In present study we investigated the role of α7nACHR in modulation of heart rate dynamics in endotoxemic rats.

## Materials and Methods

### Ethics statement

All animal maintenance and procedures were in accordance with recommendations established by the Animal Ethics Committee of Tarbiat Modares University as well as the United States NIH guidelines (publication no. 85-23). The protocol was approved by the Ethics Committee of Tarbiat Modares University. All surgeries were performed under deep anesthesia, and all efforts were made to minimize suffering.

### Chemicals

Endotoxemia was induced by intraperitoneal (IP) injection of lipopolysaccharide (LPS; *Salmonella typhimurium*) which was purchased from Sigma-Aldrich (Pool, UK). PHA-543613 hydrochloride (PHA) and methyllycaconitine citrate (MLA) were purchased from Tocris (Bristol, UK) and were used as selective agonist and antagonist for α7nACHR respectively. All of these substances were dissolved in 0.9% saline. All other reagents were purchased from Merck (Darmstadt, Germany) unless stated otherwise. 

### Animals

Male Sprague-Dawley rats were obtained from Razi Institute (Hesarak, Iran). The effect of endotoxin on heart rate dynamics was assessed following IP administration of LPS at two doses of 0.1 and 1 mg/kg. To assess the effect of α7nACHR inhibition on HRV, either saline or LPS (0.1 mg/kg) was injected 30 min after MLA administration (5 mg/kg, IP) [22]. The effect of α7nACHR activation on heart rate dynamics was evaluated by subcutaneous injection of PHA (1 or 4 mg/kg) [23,24] 30 min before IP administration of either control or LPS (1 mg/kg). 6-9 rats were used in each group. 

Our pilot studies showed that while IP administration of 1 mg/kg of LPS reduced HRV, low dose (0.1 mg/kg) LPS was unable to reduce HRV indices in rats. Based on pilot studies, 1 mg/kg of LPS was the dose that could induce maximum reduction in HRV indices. We observed that higher doses of LPS (e.g. 10 and 20 mg/kg) could not further reduce HRV in rats. We took advantage of this dose-dependent effect of LPS on HRV parameters and chose lower dose of LPS (0.1 mg/kg) for challenge with MLA (α7nACHR antagonist). This enabled us to monitor the effect of MLA on HRV when there is enough capacity for further reduction in HRV indices. Likewise, higher dose of LPS (1 mg/kg) was used to test the effect of PHA (an α7nACHR agonist) on HRV, when there is enough room to restore the loss of HRV parameters after endotoxin challenge.

### Telemetric recording of electrocardiogram and body temperature from conscious rats

Telemetric recording of electrocardiogram was carried out as described [9,11]. In brief, a dorsally mounted radiofrequency transmitter were implanted subcutaneously (lead I) in animals (body weight 230–250 g) under anesthesia using ketamine (100 mg/kg) and xylazine (10 mg/kg). 14 days after the operation, electrocardiogram (ECG) and body temperature were recorded using a telemetry system (Data Sciences International, St. Paul, Minnesota, USA) connected to a Powerlab data acquisition system (ADInstruments, Sydney, Australia).

### Data acquisition

The R peaks were detected and the R-R interval series were generated using Chart 5 software (ADInstruments). The R-R interval series were visually inspected and 5 minute artifact-free continuous R-R intervals were chosen for analysis.

### HRV analysis

The standard deviation of the R-R intervals (SDNN) was calculated on the selected artifact-free trace and used as a measure of total HRV. Non-linear measures of HRV provide information on the structure or complexity of the R-R time-series. In the present study non-linear measures of HRV were assessed using Poincaré plot, sample entropy (SampEn) as well as detrended fluctuation analysis (DFA).

#### Poincaré plot

The Poincaré plot is a graphical representation of the correlation between consecutive R-R intervals. The standard deviation of the points perpendicular to the line of identity (SD1) describes short-term variability which is mainly related to the effects of respiration on vagal drive [25]. This parameter was calculated as described [26] using the software developed by Niskanen et al. [27].

#### SampEn

SampEn was developed by Richman and Moorman in 2000 [28] and calculates the probability that epochs of window length *m* that are similar within a tolerance *r* remain similar at the next point [28]. A lower value of SampEn reflects a higher degree of regularity, and the higher the entropy value, the more random the time series is. In the present study, the parameter *m* was fixed to 2, and tolerance level *r* was 0.2 as described [9].

#### DFA

DFA quantifies fractal-like correlation properties of R-R intervals [29]. In this method, the fluctuation of the integrated and detrended data is measured within observation windows of various sizes and then plotted against window size on a log-log scale. A linear relationship between log (fluctuation) and log (window size) indicates the presence of scaling which serves as a characteristic of a fractal-like time-series [29]. The scaling exponent alpha (α) indicates the slope of this line. An α=0.5 indicates white noise (uncorrelated random data). An α greater than 0.5 and less than or equal to 1.0 indicates persistent long-range power-law correlations in which a large fluctuation is more likely to be followed by another large fluctuation. A special case α=1 corresponds to 1/f noise. When 1<α<1.5 correlation exists but cease to be of a power-law form. α=1.5 indicates the integration of white noise (Brown noise).

### Assessment of heart rate dynamics in spontaneously beating isolated heart

In order to assess the effect of LPS with or without α7nACHR agonist/antagonist on cardiac pacemaker dynamics, isolated perfused hearts were used as a model. Three hours after LPS injection, animals were anesthetized with sodium thiopental (50 mg/kg, IP). The chest wall was then opened and cardiac tissue was separated from surrounding tissues. Hearts were cannulated for retrograde perfusion according to the Langendorff method with physiological salt solution. The composition of physiological salt solution was as follows in mM: NaCl, 112; KCl, 5; CaCl_2_, 1.8; MgCl_2_, 1; NaH_2_PO_4_, 0.5; KH_2_PO_4_, 0.5; NaHCO_3_, 25; glucose, 10; and EDTA, 0.004. The solution was oxygenated with 95% O_2_ and 5% CO_2_. The temperature of the perfusate was monitored and kept at 37.0 ± 0.2 °C. In order to record spontaneous electrical activity, two stainless steel electrodes were put on right ventricle and the left ventricle. A third electrode from cannules that perfused the heart was used as reference (earth) electrode. To avoid artifact evoked by dissection, a stabilization period of 30 min was allowed before evaluation of the spontaneous electrical activity. The signals were then digitized at the sampling rate of 10 kHz and displayed on a Powerlab system (ADInstruments, Sydney, Australia). Beating rate variability parameters (SDNN, SampEn and DFA) were calculated in 10 min R-R time-series as described above.

### Expression of α7nACHR in cardiac tissue

Reverse transcription-polymerase chain reaction (RT-PCR) and immunohistochemistry were used to study the expression of α7nACHR in cardiac tissue. At least 4 separate samples were used in this part of the study.

#### RT-PCR

The heart was isolated and divided to three parts: left auricle, nodal region (atrium) and left ventricle. Samples were kept at -80 °C until RNA extraction. In addition of cardiac tissues, in order to investigate the expression of α7nACHR in isolated cardiomyocytes, H9c2 cells (rat neonate cardiomyocyte cell line) were used. The cells were purchased from Iran branch of Pasteur Institute (NCBI Code: C585). H9c2 cells were cultured in D-MEM (+ 10% fetal bovine serum) and confluent cells were detached from the culture flask using trypsin and kept frozen at -80 °C. Total RNA was extracted using RNeasy fibrous tissue mini kit (Qiagen, Germany) following the manufacturer's instructions. First strand cDNA was generated using reverse transcriptase and PCR were performed using selective forward and reverse primer for β-actin (housekeeping gene) and α7nACHR. The sequences of primers are presented as follow:

Rat α7nACHR, forward: 5’-CCTGGCCAGTGTGGAG-3’, reverse: 5’-TAAGCAAAGTCTTTGGACAC-3’ (gene accession number: NM_012832.3, product size: 414 bp). Rat β-actin, forward: 5’-AGAGGGAAATCGTGCGTGACA-3’, reverse: 5’-ACATCTGCTGGAAGGTGGACA-3’ (gene accession number: NM_031144, product size: 453 bp). 

To amplify the cDNA, PCR included incubation at 95°C for 5 min, followed by 40 cycles of thermal cycling (60 s at 95°C, 60 s at 60°C, and 60 s at 72°C). The final cycle was followed by a 5-min extension step at 72°C. PCR products were subsequently electrophoresed on 1 % agarose gel and visualized by UV lamp.

#### Immunohistochemistry

We used immunohistochemistry technique for localization of α7nACHR in rat atria. Immunohistochemistry was performed on paraffin sections (5 μm) as described [30]. The primary antibody was rabbit anti-rat α7nACHR antibody (1:700 dilution, Abcam, MA, USA), and the second antibody was biotinylated goat anti-rabbit (Bioidea, Tehran, Iran). HRP-linked streptavidin was used for staining of the immune complex. Sections were photographed by Olympus BX51 microscope with a DP27 digital camera.

### Statistical analysis

The results are presented as means ± SEM. Student’s t-test was used for comparison of two groups. Two-way ANOVA was applied when the effect of two independent variables (e.g. time after injection and the type of treatment) was assessed on a dependent variable (e.g. an HRV index). Bonferroni’s *post-hoc* test was used for comparison HRV indices at determined times. P-values less than 0.05 were considered statistically significant.

## Results

### *In vivo* study

**Figure 1:**
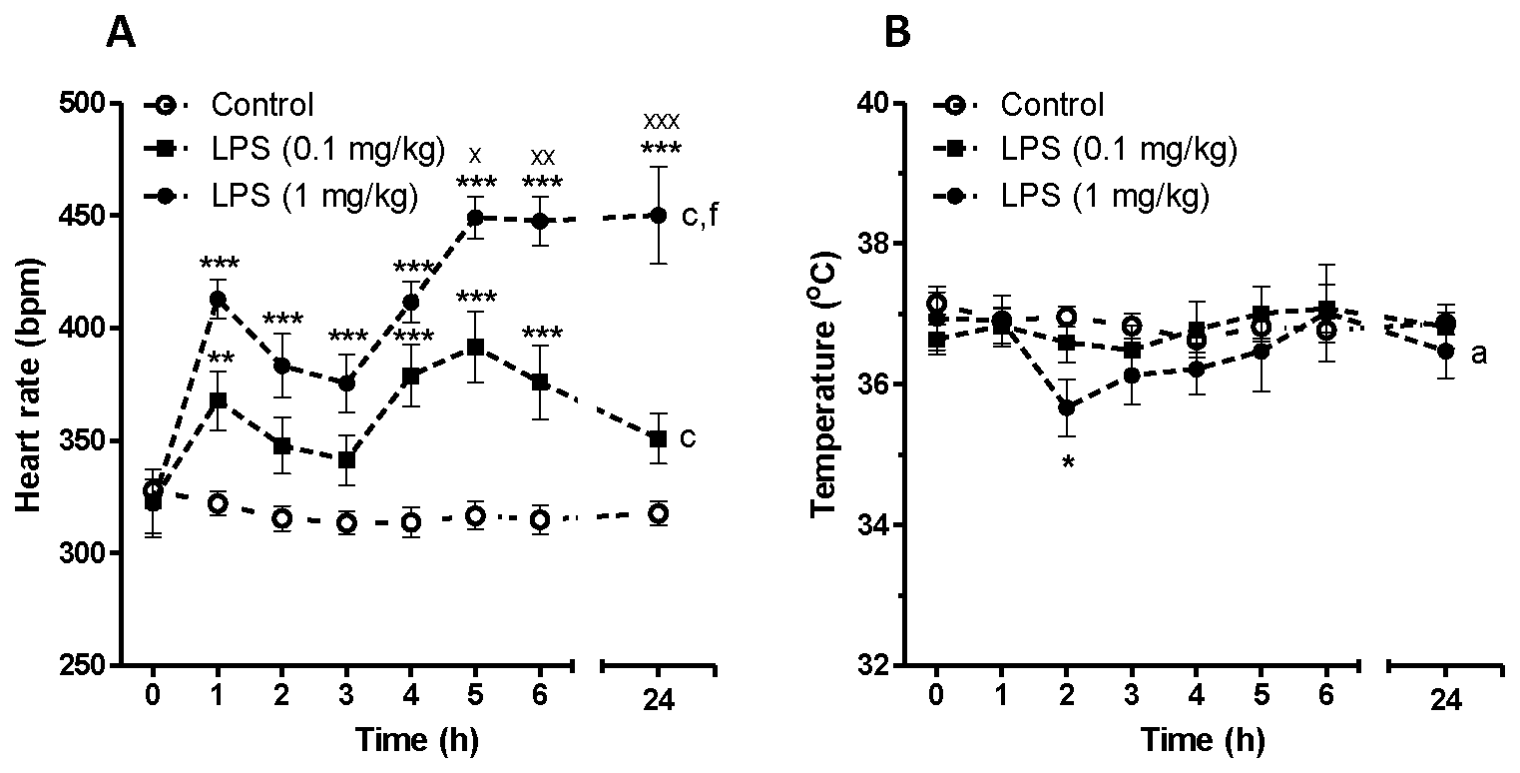
Time dependent effect of saline (control) or LPS on heart rate and body temperature in conscious rats. Data are shown as Mean ± SEM. a P<0.05, c P<0.001 (two-way ANOVA) compared to control group, f P<0.001 (two-way ANOVA) in comparison with LPS (0.1 mg/kg) group. * P<0.05, ** P<0.01 and *** P<0.001 (Bonferroni’s posttest) in comparison with control group. x P<0.05, xx P<0.01, xxx P<0.001 (Bonferroni’s posttest) compared to LPS (0.1 mg/kg) treated animals.

**Figure 2:**
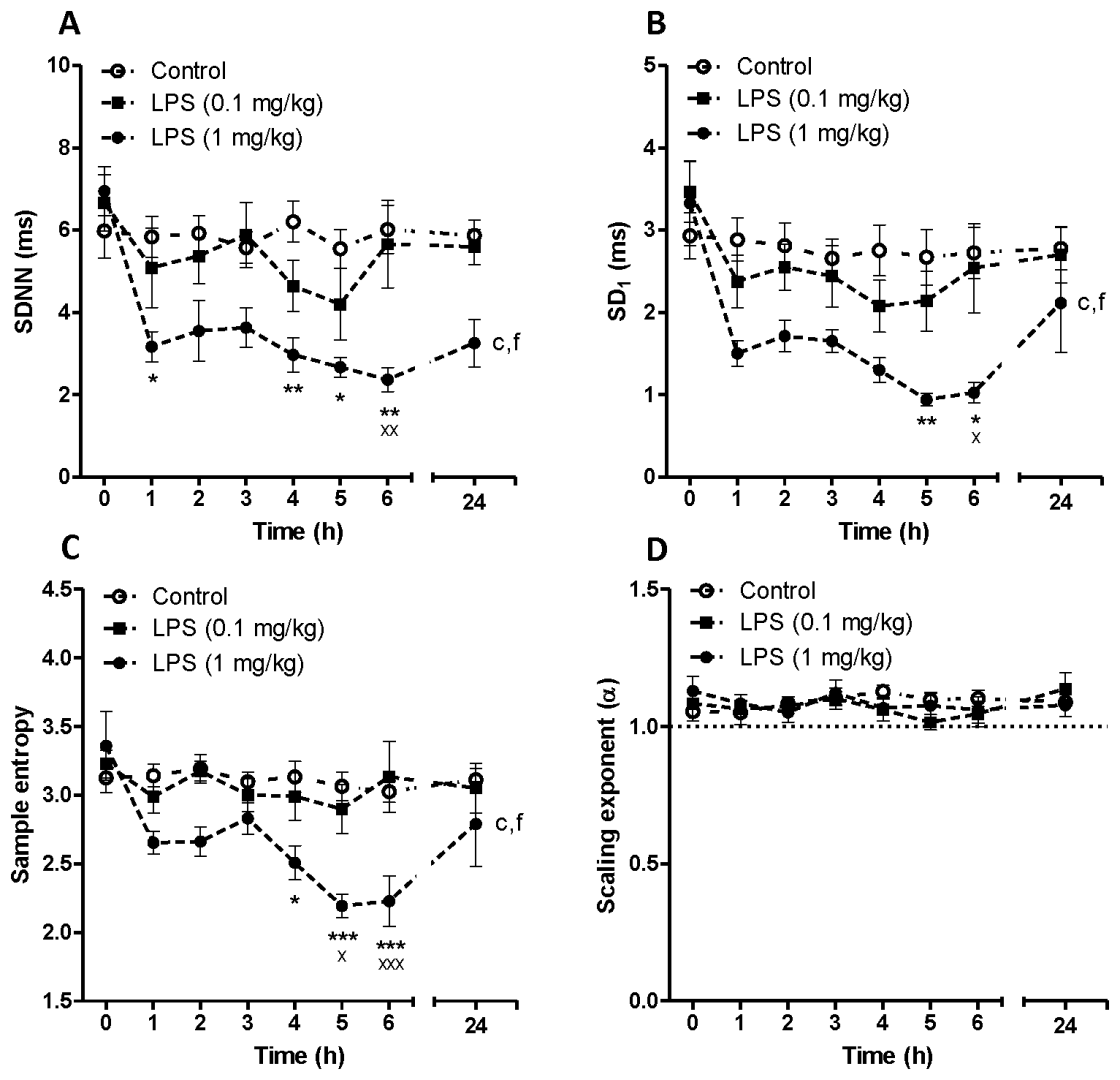
Time dependent effect of saline (control) or LPS on SDNN, SD_1_, Sample entropy and scaling exponent (α) in conscious rats. Data are shown as Mean ± SEM. c P<0.001 (two-way ANOVA) compared to control group, f P<0.001 (two-way ANOVA) in comparison with LPS (0.1 mg/kg) group. * P<0.05, ** P<0.01 and *** P<0.001 (Bonferroni’s posttest) in comparison with control group. x P<0.05, xx P<0.01, xxx P<0.001 (Bonferroni’s posttest) compared to LPS (0.1 mg/kg) treated animals.

We initially assessed the effect of two doses of LPS on heart rate dynamics in conscious rats using a telemetric device. The effect of α7nACHR antagonist or agonist was then assessed in this rat model. Figure 1 shows time-dependent effect of LPS (0.1 mg/kg and 1 mg/kg) on heart rate and body temperature. Endotoxin injection was associated with a biphasic increase in heart rate at both given doses of LPS. The effect of LPS on heart rate was statistically significant according to two-way ANOVA (F_LPS 0.1 mg/kg vs control_ = 77.85, P<0.001, F_LPS 1 mg/kg vs control_ = 386.5, P<0.001, Figure 1A). Low dose of LPS (0.1 mg/kg) was unable to change body temperature within 24 h post-LPS injection. However, higher dose of LPS (1 mg/kg) was able to induce a transient hypothermia in rats as shown in Figure 1B (F_LPS 1 mg/kg vs control_ = 6.46, P<0.05). Figure 2 indicates the effect of LPS on HRV parameters *in vivo*. Low dose LPS (0.1 mg/kg) did not significantly decrease HRV indices including SDNN, SD1, SampEn and scaling exponent (Figure 2). LPS at higher does (1 mg/kg), however, could significantly reduce SDNN (Figure 2A), SD1 (Figure 2B) and SampEn (Figure 2C) as assessed by two-way ANOVA as well as *post-hoc* tests. The effect of acute endotoxin challenge on fractal-like structure of R-R time series was also investigated using DFA. There was linear relationship between log (fluctuation) and log (window size) in both control and LPS-treated groups at any given time-scale. The scaling exponent (α) was 1.055 ± 0.034 which corresponds to fractal-like structure with 1/f dynamics [29]. LPS injection at both high and low dose did not change this type of dynamics within 24 h telemetric study.

**Figure 3:**
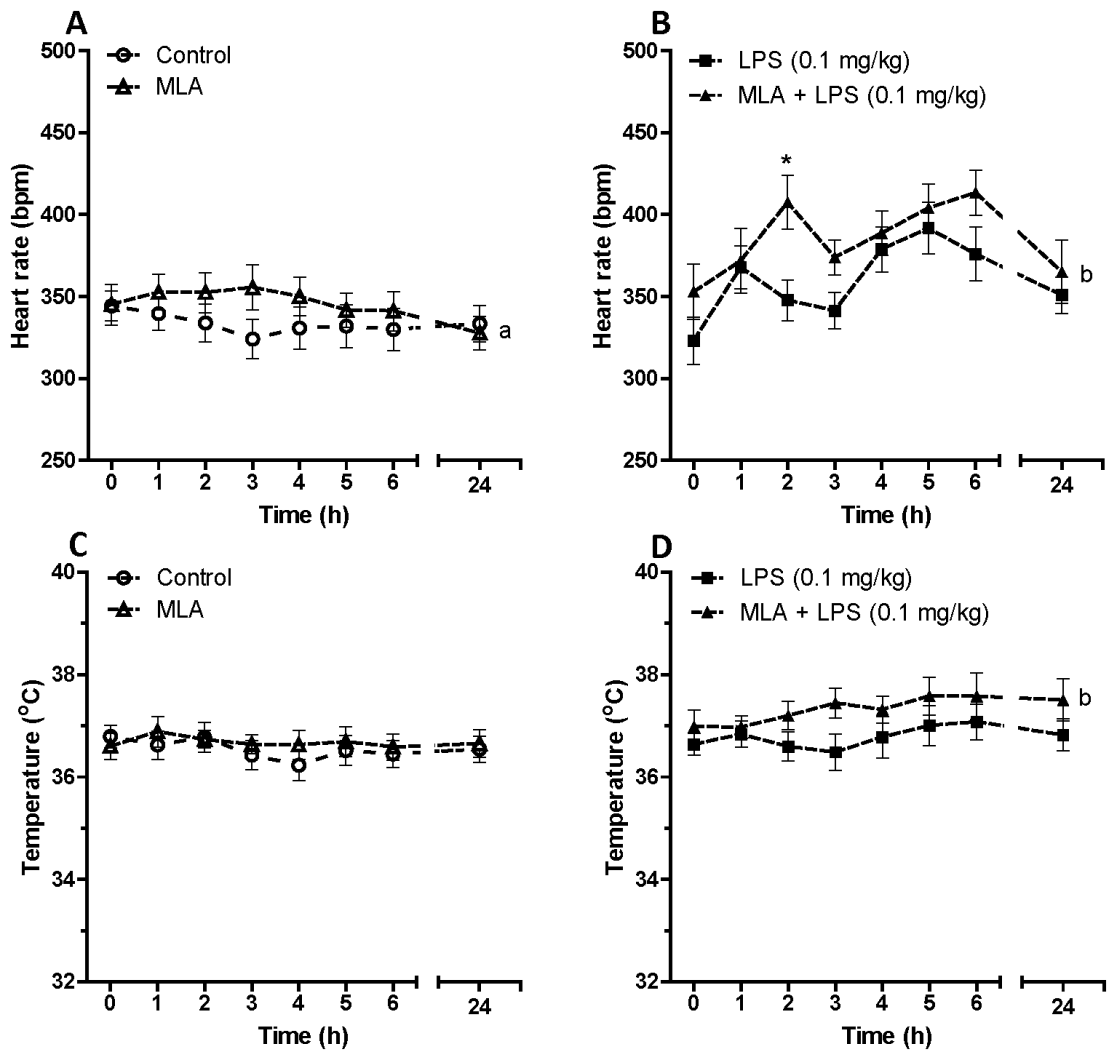
Time dependent effect of an α7nACHR antagonist (methyllycaconitine citrate) on heart rate and body temperature in conscious rats given saline or LPS (0.1 mg/kg). Data are shown as Mean ± SEM. MLA: methyllycaconitine citrate. a P<0.05 (two-way ANOVA) in comparison with control group, b P<0.01 (two-way ANOVA) compared to LPS-treated group. * P<0.05 (Bonferroni’s posttest) in comparison with LPS-treated group.

We then looked at the effect of pre-treatment with either an α7nACHR antagonist (MLA) or agonist (PHA) on heart rate dynamics and body temperature in naïve and endotoxemic conscious rats. Figure 3 shows the effect of MLA on heart rate and body temperature in saline or LPS (0.1 mg/kg) treated rats. MLA induced a transient but negligible increase in heart rate in control rats as shown in Figure 3A. This effect on heart rate was significantly exaggerated after low dose of LPS (Figure 3B). There was a statistically significant elevation of heart rate in MLA + LPS (0.1 mg/kg) treated group in comparison with LPS (0.1 mg/kg) treated rats (F_MLA + LPS vs. LPS_=10.88, P<0.01, two-way ANOVA). Figure 3C and 3D shows that α7nACHR blockade was unable to alter body temperature in controls rats but could increase body temperature in endotoxemic animals (F_MLA + LPS vs. LPS_=10.42, P<0.01, two-way ANOVA).

**Figure 4:**
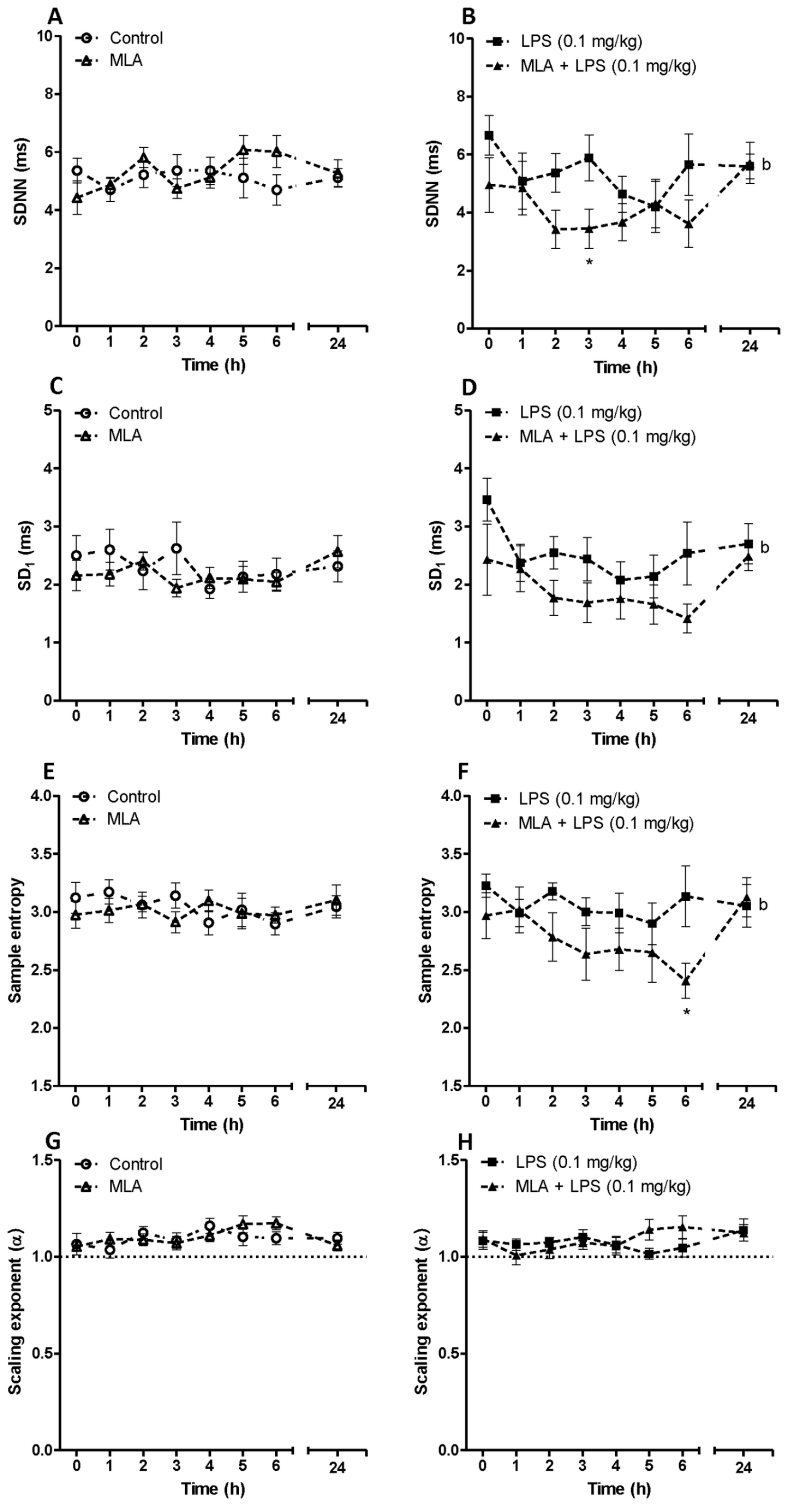
Time dependent effect of an α7nACHR antagonist (methyllycaconitine citrate) on SDNN, SD_1_, Sample entropy and scaling exponent in conscious rats given saline or LPS (0.1 mg/kg). Data are shown as Mean ± SEM. MLA: methyllycaconitine citrate. b P<0.01 (two-way ANOVA) compared to LPS-treated group. * P<0.05 (Bonferroni’s posttest) in comparison with LPS-treated group.

Figure 4 exhibits the effect of MLA on HRV parameters *in vivo*. MLA administration did not induce any significant effect on SDNN, SD1 or SampEn in saline-treated rats (Figure 4A, 4C and 4E). However, MLA pre-treatment in endotoxemic rats was associated with a reduction in SDNN, SD1 and SampEn within the first 6 h after LPS injection which was statistically significant according to two-way ANOVA (F_MLA + LPS vs LPS_ = 6.4, P<0.01; F_MLA + LPS vs LPS_ = 7.79, P<0.01; F_MLA + LPS vs LPS_ = 7.34, P<0.01 for SDNN, SD1 and SampEn respectively). Acute α7nACHR blockade did not exhibit any significant effect on fractal-like structure or the scaling exponent in neither control nor endotoxemic group (Figure 4G and 4H).

**Figure 5:**
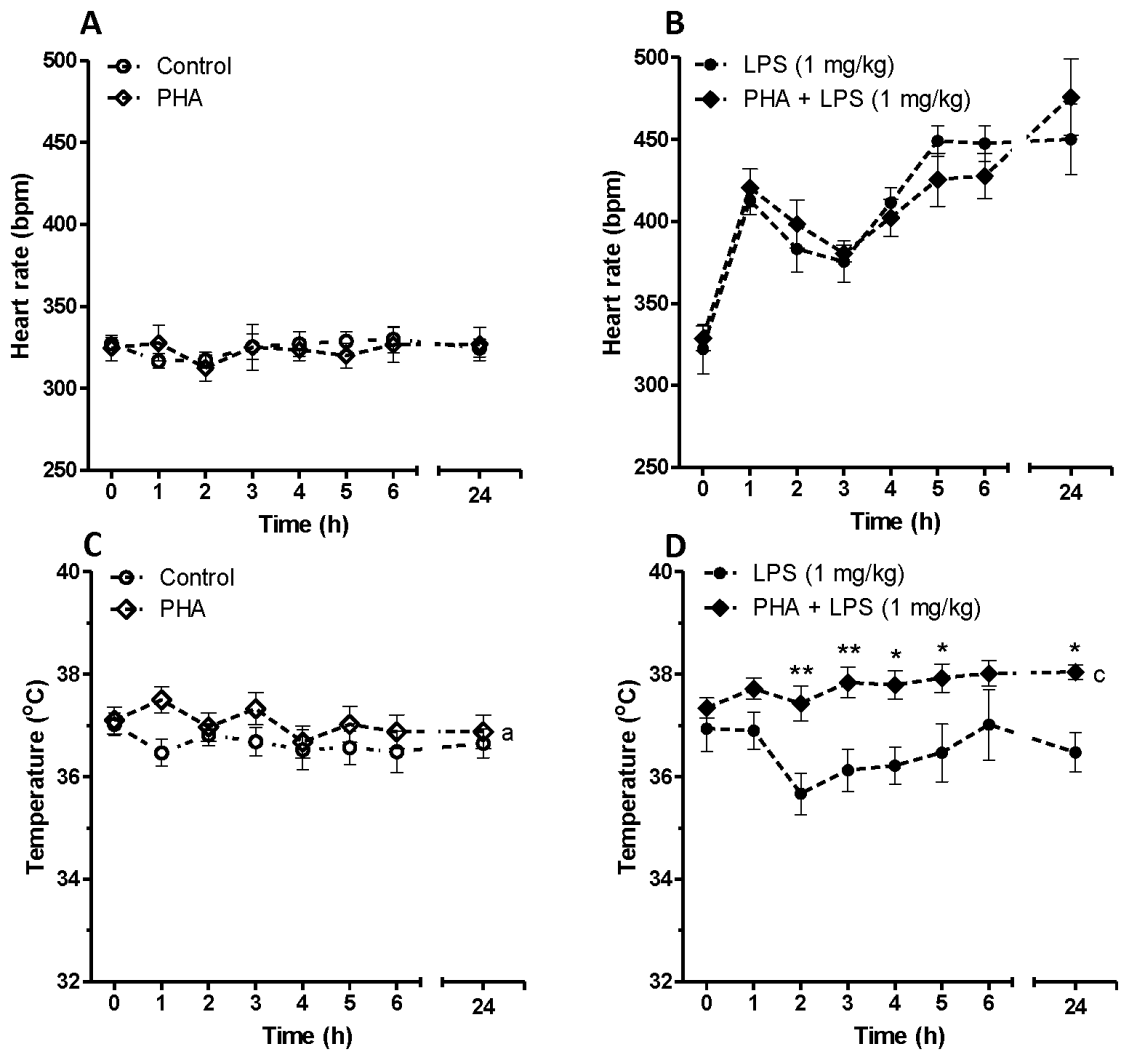
Time dependent effect of an α7nACHR agonist (PHA-543613) on heart rate and body temperature in conscious rats given saline or LPS (1 mg/kg). Data are shown as Mean ± SEM. PHA: PHA-543613. a P<0.05 (two-way ANOVA) in comparison with control group, c P<0.001 (two-way ANOVA) compared to LPS-treated group. * P<0.05, ** P<0.01 (Bonferroni’s posttest) in comparison with LPS-treated group.

**Figure 6:**
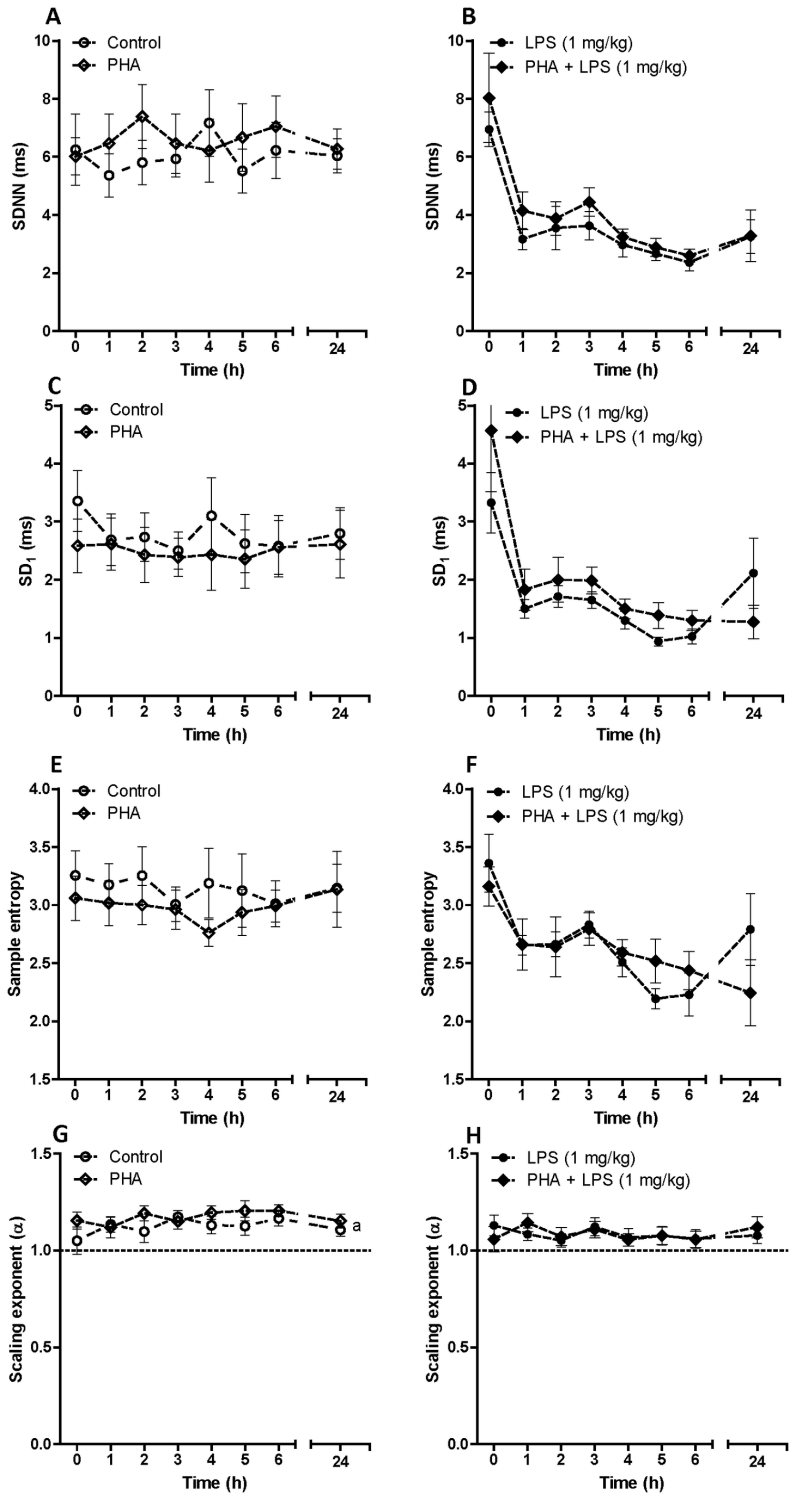
Time dependent effect of an α7nACHR agonist (PHA-543613) on SDNN, SD1, Sample entropy and scaling exponent (α) in conscious rats given saline or LPS (1 mg/kg). Data are shown as Mean ± SEM. PHA: PHA-543613. a P<0.05 (two-way ANOVA) in comparison with control group.

The effect of an α7nACHR agonist (PHA) on heart rate dynamics and body temperature was assessed in naïve and endotoxemic rats (Figure 5 and 6). Pre-treatment with PHA was unable to affect heart rate in conscious animals given saline or LPS (1 mg/kg) (Figure 5A and 5B). PHA administration, however, exhibited a significant effect on body temperature particularly in LPS-treated rats (Figure 5C and 5D). LPS (1 mg/kg) administration was associated with a transient hypothermia and this was reversed with pre-treatment with PHA as shown in Figure 5D (F_PHA + LPS vs LPS_= 36.28, P<0.001, two-way ANOVA). We assessed the effect of PHA administration on SDNN, SD1, SampEn and scaling exponent and none of these parameters were altered with PHA administration in endotoxemic rats (Figure 6).

### *Ex vivo* study

In *ex vivo* experiment the rat hearts were isolated 3 hours after LPS or saline injection. Mean heart rate, SDNN, SampEn and scaling exponent were calculated and presented in Table 1. Mean heart rate of isolated heart in control rats were 312 ± 8 bpm which was similar with the heart rate in couscous rats (316 ± 5 bpm; *in vivo* data). We observed a significant reduction in SDNN when *in vivo* data were compared with *ex vivo* data in control animals (5.61 ± 0.37 ms *vs* 3.54 ± 0.33 ms, P<0.001). The same phenomenon was observed for SampEn (3.10 ± 0.07 *vs* 0.53 ± 0.10 *in vivo* and *ex vivo* study respectively, P<0.001). This indicates that denervation of cardiac pacemaker reduces heart rate variability which is a known phenomenon. We also looked at fractal-like dynamics in *ex vivo* data and observed a linear relationship between log (variation) and log (scale) using DFA in all experimental groups. Scaling exponent (α) was 1.29 ± 0.02 in isolated heart of control rats which was comparable with this parameter in conscious animals (1.055 ± 0.034, P<0.001). 

**Table 1:** Mean heart rate and HRV indices in *ex vivo* experiment.

	Groups						
Index	Control	LPS 0.1 mg/kg	LPS 1 mg/kg	PHA	MLA	PHA + LPS 1 mg/kg	MLA + LPS 0.1 mg/kg
Heart rate (bpm)	312 ± 8	312 ± 7	297 ± 8	307 ± 11	316 ± 5	280 ± 7	298 ± 9
SDNN (ms)	3.54 ± 0.33	3.96 ± 0.31	4.33 ± 0.74	3.42 ± 0.90	3.31 ± 0.13	4.93 ± 0.90	5.68 ± 1.47
SampEn	0.53 ± 0.10	0.37 ± 0.13	0.57 ± 0.11	0.43 ± 0.13	0.29 ± 0.05	0.44 ± 0.2	0.29 ± 0.06
Scaling exponent	1.29 ± 0.02	1.31 ± 0.04	1.32 ± 0.04	1.32 ± 0.05	1.34 ± 0.02	1.32 ± 0.07	1.34 ± 0.05

Data are shown as Mean ± SEM. There is no statistically significant difference between control and endotoxemic groups in mean heart rate, SDNN, SampEn or scaling exponent. Pre-treatment with α7nACHR antagonist (MLA) or agonist (PHA) did not show any significant difference in mean heart rate and HRV indices.

As show in Table 1, there was no significant difference between naïve and endotoxemic groups in mean heart rate, SDNN, SampEn or scaling exponent. Likewise, pre-treatment with α7nACHR antagonist or agonist did not exhibit any significant difference in mean heart rate and HRV indices in *ex vivo* groups (Table 1).

### Detection of α7nACHR in cardiac tissue

**Figure 7:**
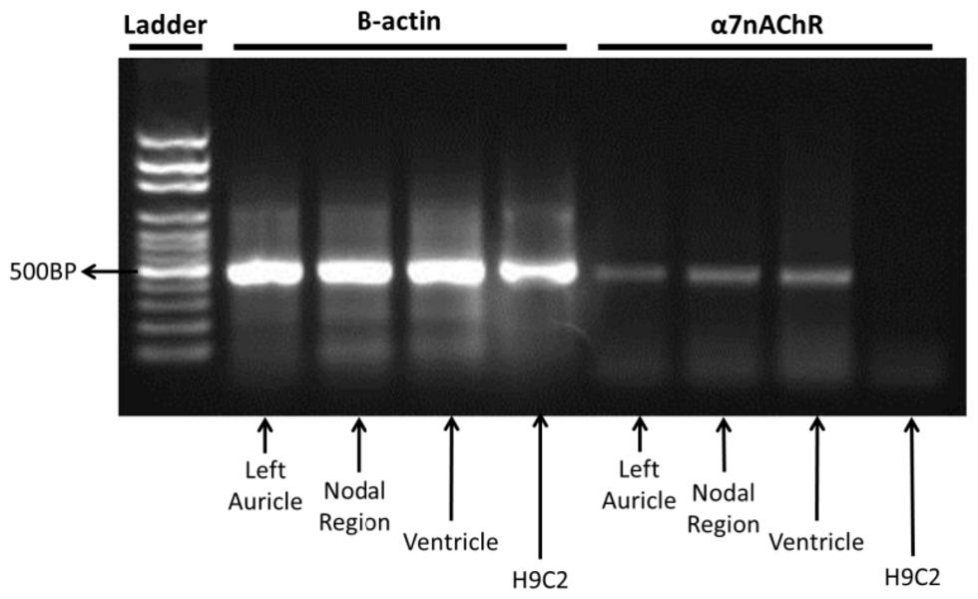
Expression of β-actin and α7nACHR in rat left auricle, atrium (nodal region), left ventricle and H9c2 cells. RNA was isolated and analyzed by RT-PCR.

RT-PCR was used to detect the expression of α7nACHR in rat cardiac tissue as well as H9c2 cells (rat cardiomyocyte). Figure 7 shows the results of RT-PCR for β-actin (housekeeping) and α7nACHR. All samples expressed β-actin. α7nACHR was detectable in the atrium (left auricle and nodal region) and left ventricle expressed with corresponding PCR product of ~ 414 bp (Figure 7). We were unable to find evidence for expression of α7nACHR in H9c2 cells as shown in Figure 7. This data was reproducible in at least 4 separate samples.

**Figure 8:**
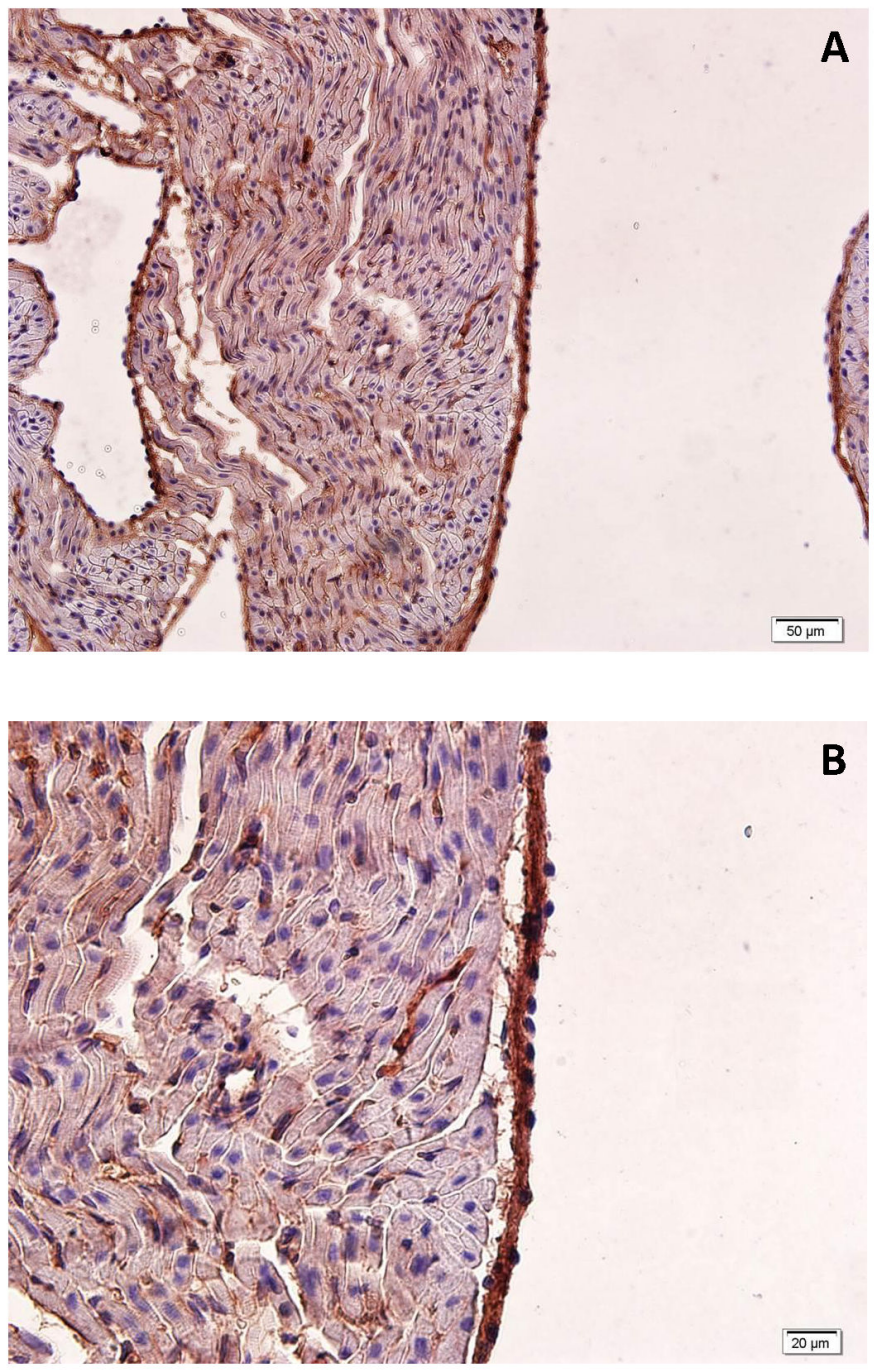
Immunohistochemistry study using anti α7nACHR antibody in rat left auricle. A. X 200, B. X 400 magnification. Tissue sections and immune complexes were stained using hematoxylin and diaminobenzidine (DAB) respectively.

Immunohistochemistry was employed for tissue localization of α7nACHR in left auricle using anti-α7nACHR antibody. Strong immunostaining could be observed in endothelial cells as shown in Figure 8. Although scattered immunostaining was also visible in other parts of the left auricle, almost no immunostaining could be seen in the myocardial layer. In same samples, we omitted primary antibody from the staining protocol and did not observe non-specific staining with the secondary antibody (data not shown).

## Discussion

Autonomic dysfunction and loss of HRV are common complications of systemic inflammation which has both diagnostic and prognostic value in a variety of diseases such as sepsis, diabetes and cirrhosis [4,26,31,32]. Since the discovery of α7nACHR expression in inflammatory cells, many investigators have reported that these receptors can modulate systemic inflammation in different model systems [33]. The present study was aimed to test the hypothesis that α7nACHR can modulate loss of HRV during systemic inflammation. We used endotoxemia as an animal model of systemic inflammatory response syndrome (SIRS) and confirmed that endotoxin can affect heart rate and its variability in conscious rats. In present study different doses of LPS were used. The higher dose (1 mg/kg) was able to induce biphasic tachycardia, transient hypothermia as well as a significant reduction in HRV parameters. This finding corroborates with our previous reports in conscious rats [9,11]. The lower dose (0.1 mg/kg) could induce tachycardia but failed to reduce HRV or change body temperature within 24 h. It appears that low dose LPS (0.1 mg/kg) is able to induce a slight increase in pro-inflammatory cytokines in rat [34], however, this slight increase in inflammatory mediators was not enough to reduce HRV or induce fever in this animal model. We took advantage of this dose-dependent effect of LPS on HRV parameters and chose lower dose of LPS for challenge with MLA (α7nACHR antagonist). This enabled us to monitor the effect of MLA on HRV when there is enough capacity for further reduction in HRV indices. Likewise, higher dose of LPS (1 mg/kg) was used to test the effect of PHA (an α7nACHR agonist) on HRV, when there is enough room to restore the loss of HRV parameters after endotoxin challenge.

Our data showed that although low dose LPS was unable to reduce HRV indices, pharmacological inhibition of α7nACHR could significantly reduce SDNN, SD1 and SampEn in conscious rats after low dose LPS challenge. Acute administration of MLA did not exhibit a significant effect on HRV parameters in naïve rats. This finding indicates that α7nACHR modulates heart rate dynamics during endotoxemia; a phenomenon which has not been reported before. The lack of a significant effect of MLA on HRV parameters in naïve rats goes along with the recent report by Deck et al. who showed that α7nACHR is not required for parasympathetic control of heart in mouse [20]. α7nACHR deficient mice exhibit normal short-term and long-term HRV in comparison with wild type mice [20]. Therefore the effect of MLA on heart rate dynamics in endotoxemic rats might be due to interaction of α7nACHR signaling with endotoxin-related mechanisms (e.g. potentiation of systemic inflammation). A large body of evidence supports that nicotine suppresses LPS-induced inflammation through α7nACHR [35–38]. This interaction has been reported at different mechanistic levels; for instance, Hamano et al. found that α7nACHR activation suppresses the expression of toll-like receptor 4 (TLR4) on monocytes and inhibits the production of TNF-α in human peripheral blood mononuclear cells in the presence of LPS [39]. Other studies have shown that α7nACHR activation may inhibit TLR4-dependent mechanisms via suppression of I-kappaB phosphorylation [40]. Overall, these reports provide evidence for the existence of an α7nACHR-dependent anti-inflammatory pathway.

We used linear and non-linear methods for assessment of cardiac cycle variability. SDNN is a linear index of heart rate fluctuation. SD1 decomposes short-rate HRV and SampEn gives information on the degree of regularity of cardiac cycles. MLA could decrease all these indices in endotoxemic rats indicating that not only total variability of cardiac cycle is reduced but MLA + LPS rats showed more regular cardiac rhythm in comparison with LPS treated rats. According to Pincus [41] loss of entropy in an interconnected system is a hallmark of system isolation. Thus, α7nACHR inhibition may induce partial isolation of the regulatory components in rats given low dose LPS. Gholami et al. reported that endotoxemia in rats is associated with partial uncoupling of cardiac pacemaker from cholinergic neural control [9]. This phenomenon has been described in other models [10,11] and seems to be a reason for system isolation and reduced entropy of cardiac rhythm during systemic inflammation. We did not directly measure pacemaker responsiveness to a parasympathomimetic following MLA + LPS administration. This study can be carried out in future. SD1 is an index for short-term variability and is mainly mediated by vagal activity [25]. A significant reduction in SD1 shows that either vagal activity or the pacemaker responsiveness to parasympathetic control is impaired in MLA + LPS (0.1 mg/kg) group, a phenomenon that was not observed in rats given LPS (0.1 mg/kg) alone.

It is well known that physiological rhythms such as heart rate have a fractal temporal structure [9,29]. It seems that the fractal-like scaling behavior is robust and remains unaffected by small perturbation of physiological system [9,11]. Ivanov et al. showed changes in fractal-like dynamics of cardiac rhythm in patients with a life-threatening condition such as heart failure [42]. Haddadian et al. also reported that although fractal-like behavior of HRV remains unchanged by sub-lethal doses of endotoxin, lethal dose is associated with a significant decrease in scaling exponent [11]. We used low doses of LPS in our present study which is much lower than LD_50_ of LPS in rats (22 mg/kg) [43]. We did not observe a significant alteration in fractal dynamics in endotoxemic rats given MLA or PHA. This confirms the robust nature of fractal-like structure of cardiac cycles in response to sub-lethal doses of endotoxin.

It is well known that HRV represents the complex interaction between autonomic nervous system and cardiac pacemaker. Although the majority of variations in cardiac rhythm are related to fluctuations in autonomic nervous system, pacemaker dynamics itself plays a role in complexity of cardiac rhythm [9,44]. Our data showed that beating variability of the *ex vivo* study was much less than *in vivo* study (as assessed by SDNN and SampEn). However, both denervated (*ex vivo*) and innervated (*in vivo*) hearts showed fractal-like dynamics. This indicates that pacemaker rhythm itself has a complex dynamics and the interaction between autonomic control mechanism and pacemaker increases variability and entropy of the system. Previous studies have shown that both autonomic nervous system and cardiac pacemakers can be targets for inflammatory mediators. There is evidence to show that beating dynamics of denervated cardiac pacemaker is altered during endotoxemia [9,45]. Schmidt et al. showed decreased beating rate variability of spontaneously contracting cardiomyocytes after incubation with endotoxin [45]. The same group of investigators also reported impaired pacemaker current (I_*f*_) after incubation with LPS in human cardiomyocytes [46]. In order to see whether or not the effect of α7nACHR blockade is related to alteration in pacemaker dynamics, we assessed HRV parameters in isolated perfused hearts in the experimental groups. The results showed that administration of endotoxin was unable to change either beating rate, SDNN, SampEn or scaling exponent in denervated hearts. Moreover, neither MLA nor PHA could make a significant effect on HRV parameters *ex vivo*. This is in line with autonomic neuropathy or impaired pacemaker responsiveness to autonomic control in endotoxemic rats.

Endotoxin is known to induce dose-dependent paradoxical changes in body temperature in mammals [47]. Low grade systemic inflammation is associated with fever, while high doses of endotoxin may cause hypothermia [47]. Our data showed that while LPS (1 mg/kg) significantly reduced body temperature within first 3 h of administration, the lower dose (0.1 mg/kg) did not change body temperature within 24 h post-LPS injection. Acute α7nACHR blockade was associated with low grade fever in rats given non-pyrogenic dose of LPS (0.1 mg/kg) and this finding goes along with enhanced systemic inflammation in endotoxemic rats given MLA. The effect of α7nACHR on body temperature was not the focus of our study; therefore we did not perform telemetric study in neutral ambient temperature. Thermoregulation studies in rats have historically been with controversy within context of different febrile phases in response to endotoxin challenge [48]. However, it was found that the febrile phase can be readily overlooked when the ambient temperature is below neutral temperature. Our telemetry studies were conducted in rats housed in their home cages at room temperature, which is normally sub-neutral for this species [48]. Therefore we might have missed a febrile phase within our experimental setting. Our data showed that α7nACHR inhibition is associated with prolonged low grade fever in endotoxemic rats and not naïve animals. This data can be confirmed in neutral ambient temperature in future studies. It is logical to see an increase in heart rate followed by increased body temperature and reduced vagal activity (as assessed by SD1). This was what we observed when the heart rate of LPS-treated rats was compared with endotoxemic rats given MLA.

Although α7nACHR blockade could modulate heart rate and its dynamics in endotoxemic rats, PHA (α7nACHR agonist) did not induce any significant change in either heart rate or HRV parameters in LPS-treated animals. Pre-treatment of rats with PHA could abolish LPS-induced hypothermia. This observation is in agreement of an anti-inflammatory function for α7nACHR. However we were unable to show modulation of HRV via α7nACHR activation. We used two different doses of PHA (1 mg/kg and 4 mg/kg) and observed that heart rate, SDNN, SD1 and SampEn were not significantly affected by low dose (1 mg/kg) or high dose (4 mg/kg, data not shown) of PHA. Previous reports have shown that once cardiac cycle variability is reduced during systemic inflammation, it is difficult of restore HRV indices using classic anti-inflammatory challenges [8,21]. Fairchild et al. demonstrated that dexamethasone shortens but does not eliminate LPS-induced HRV depression in mice [8]. According to this report, pretreatment with dexamethasone significantly attenuated LPS-induced production of pro-inflammatory cytokines but had no effect on the magnitude of the initial response of depressed HRV following LPS administration [8]. Likewise Alvarez et al. reported that administration of a glucocorticoid before endotoxin reduced cytokine levels but did not affect HRV indices in human volunteers [21]. Present study also revealed that an α7nACHR agonist did not have any significant effect on heart rate dynamics in endotoxemic rats. This indicates that cytokine-induced changes in HRV is complex and once triggered it is relatively resistant to modulation. By analogy, this type of dynamics exhibits “hysteresis”, a phenomenon that has been observed in a variety of complex systems such as immune system [49]. Hysteresis in this case is used as once inflammation-induced changes on HRV are triggered, its suppression may be difficult to achieve. Apart from this hypothetical mechanism, other possible mechanisms might explain our observation. If basal α7nACHR activity exerts a tonic anti-inflammatory effect (close to its maximum capacity) in cardiovascular regulatory system, one would expect to observe that α7nACHR blockade could potentiate the effect of LPS on HRV, while an α7nACHR agonist can no longer restore LPS-induced changes on heart rate dynamics. This explanation goes along with recent report by Kox et al. who investigated the anti-inflammatory effects of GTS-21 (an α7nACHR agonist) on the innate immune response during experimental human endotoxemia [50]. In their double-blind study they showed that the highest dose of GTS-21 did not result in significant differences in inflammatory mediators between the GTS-21 and placebo-treated groups [50]. Based on our observations we suggest a tonic role for α7nACHR in modulation of inflammation in cardiovascular regulatory centers. Although these hypothetical explanations are interesting within the context of cholinergic anti-inflammatory pathway, they need to be investigated comprehensively in future.

Although the atrium receives dense innervations from the vagus nerve, the effect of this cholinergic anti-inflammatory pathway has not rigorously been investigated in the atrium. We observed that α7nACHR is expressed in rat atrium and its localization is mainly at endothelial layer. Neonatal rat cardiomyocytes in culture did not express these receptors. Likewise we were unable to see a significant immunostaining for α7nACHR in myocytes in the immunohistochemical study of adult atria. We also tested if there is any alteration in the expression of α7nACHR in rat atria upon LPS treatment (appendix 1 in File S1). The results showed that α7nAChR mRNA level did not change upon LPS treatment in rat atria (appendix 1 in File S1). In another study, we incubated H9c2 cells with LPS in culture and looked at α7nAChR expression using RT-PCR. Likewise, incubation with endotoxin was unable to induce α7nAChR expression in this cell line (appendix 1 in File S1). The expression of α7nACHR in the endothelium is an interesting finding and might suggest a role for this receptor in the cross-talk between endothelium and other cardiac cells. A cross talk between endothelium and atrial cardiomyocytes has been reported for atrial natriuretic peptide secretion from the atrium [51]. To the best of our knowledge the role of nicotinic cholinergic receptors in modulation of the cross-talk between endothelium and cardiac cells has not been investigated and such interaction can be investigated in future. Our results indicated that α7nACHR inhibition may modulate cardiac cycle variability in endotoxemic rats. This finding does not necessarily mean that endothelial α7nACHR (within atrium) is the main target for the observed effect. In order to test the hypothesis that systemic α7nACHR blockade might affect expression of pro-inflammatory mediator in the atrium, we compared atrial expression of interleukin-1 (IL-1) and monocyte chemoattractant protein-1 (MCP-1) in control and endotoxemic rats given either saline or MLA. Our results showed that although LPS (0.1 mg/kg) significantly increased both IL-1 and MCP-1 mRNA levels in rat atria, MLA was unable to change atrial levels of these pro-inflammatory mediators (appendix 2 in File S1). Based on these observations, it is less likely that MLA mediates its effects on HRV through interaction with atrial α7nACHR. α7nACHR is widely expressed in variety of cells that are known to modulate systemic inflammation [14]. Sakata et al. mapped α7nACHR in human using positron emission tomography technique and showed that the α7nACHR is mostly visible in human liver [52]. Moreover, various investigations also showed that non-neuronal cholinergic system is involved in the pathophysiology of disease [17]. In this view, neurons (e.g. vagus nerve) are not the only source of acetylcholine synthesis in mammals and a variety of endothelial, epithelial and lymphocytes can synthetize and secret acetylcholine in an autocrine or paracrine fashion [17]. This distribution of α7nACHR and acetylcholine secreting cells help us to assume that the effect of MLA on HRV parameters during systemic inflammation may involve a variety of cells and systems. Among cytokines, circulating level of IL-6 has the best correlation with HRV indices in sepsis [10]. It has also been shown that IL-6 might play a mechanistic role in reduced HRV during systemic inflammation [10]. Therefore we collected sera from healthy and endotoxemic animals (treated with Saline, MLA or PHA) and measured IL-6 levels using a commercial kit (rat IL-6 platinum ELISA, eBioscience, Vienna, Austria). Although we could see a linear standard curve for rat recombinant IL-6 (31.3-2000 pg/ml), the level of IL-6 in serum samples were below the detection limit of most commercially available ELISA kits (12 pg/ml). Therefore we could not compare serum IL-6 level between our experimental group and comparison of circulating IL-6 levels remains an open question for future investigation.

One limitation of our study is that blood pressure was not measured within our experimental setting. However, Fairchild et al. have recently showed that loss of heart rate variability following LPS challenge could not be explained by changes in blood pressure in conscious mice [8]. Moreover, continuous measurement of blood pressure in freely-moving rats required manipulation of main arteries which could potentially affect the fine dynamics of cardiovascular regulatory system. Another limitation of the present study might be related to the systemic administration of the α7nACHR antagonist (or agonist). Although we could observe a significant effect following α7nACHR blockade in endotoxemic rats, we do not know which cell (or organ) has been the target for the observed effect. Future studies using transgenic *Cre-Lox* technology can pave the way to understand the role of endothelial α7nACHR in modulation of cardiac function.

Cardiac cycle variability is a complex phenomenon which shows both deterministic and stochastic behaviors [53]. This complex behavior can be investigated using a variety of methods such as DFA. Fractal-like structure of the R-R time-series is usually robust and does not show significant alteration with manipulations. In present study we observed that the scaling exponent of R-R time-series showed a subtle and transient increase after systemic injection of PHA in healthy rats. This indicates that the scale-free variability goes towards Brown noise after α7nACHR activation [29]. We cannot explain this result with current information and this phenomenon can be investigated in later studies.

We used a pharmacological approach using systemic administration of 5 mg/kg of MLA in order to block α7nACHR in vivo. Although MLA is one of the most selective antagonists available for α7nACHR, a potential limitation of using a pharmacological antagonist is that high concentration might exhibit non-specific interaction with other nicotinic acetylcholine receptor subtypes. In order to determine whether pharmacologically relevant concentrations of MLA could be achieved in plasma following peripheral administration of MLA, Turek et al. measured plasma concentration of MLA following IP administration of MLA (6.2 µmol/kg ≈ 5.4 mg/kg) [54]. They showed that maximal plasma levels of MLA was 694 ± 106 ng/ml; a concentration that is within a range previously reported to selectively block α7nACHR mediated responses in vitro [54]. More detailed studies may confirm our findings using α7nACHR knockout mice. In fact, Deck et al. used α7nACHR deficient mice and showed that these transgenic animals exhibit normal HRV indices in comparison with wild type mice [20]. Their report goes along with our findings in naïve rats after MLA challenge. Although future studies can test the effect of endotoxin on heart rate dynamics in α7nACHR knockout mice, one limitation of using general knockout animals is that, the absence of α7nACHR expression during embryonic development might potentially induce compensatory alteration in the expression of other genes that are involved in modulation of inflammation or cardiovascular regulation. Therefore, we suggest using conditional knockout technology (e.g. using *Cre-lox* method) for further evaluation of the role of α7nACHR in modulation of heart rate dynamics during systemic inflammation. 

We investigated the effect of systemic inhibition of α7nACHR on heart rate dynamics and observed that α7nACHR blockade can modulated HRV in endotoxemic rats. This observation corroborates with the role of α7nACHR in cholinergic anti-inflammatory pathway and suggests a tonic role for nicotinic acetylcholine receptors in modulation of inflammation in cardiovascular regulatory centers during endotoxemia.

## Supporting Information

File S1 **Contains: Appendix 1: The effect of endotoxin of expression of α7nACHR in rat atria and H9c2 cells.** Appendix 2: The effect of pharmacological α7nACHR blockade on atrial expression of IL-1 and MCP-1 in rat atria.
(DOCX)
