# Correction: The Role of α_7_ Nicotinic Acetylcholine Receptor in Modulation of Heart Rate Dynamics in Endotoxemic Rats

**DOI:** 10.1371/annotation/4103a8bf-a523-4db9-91f2-d974b175aafa

**Published:** 2013-12-20

**Authors:** Roham Mazloom, Golnar Eftekhari, Maryam Rahimi, Vahid Khori, Sohrab Hajizadeh, Ahmad R. Dehpour, Ali R. Mani

Affiliation number 3 for the fourth author is incorrect. The correct affiliation is: Ischemic Disorders Research Center, Golestan University of Medical Sciences, Gorgan, Iran. 

