# Correction: The Role of α7 Nicotinic Acetylcholine Receptor in Modulation of Heart Rate Dynamics in Endotoxemic Rats

**DOI:** 10.1371/journal.pone.0127826

**Published:** 2015-05-01

**Authors:** Roham Mazloom, Golnar Eftekhari, Maryam Rahimi-Balaei, Vahid Khori, Sohrab Hajizadeh, Ahmad R. Dehpour, Ali R. Mani

The third author’s name is incorrect. The correct name is: Maryam Rahimi-Balaei. The correct citation is: Mazloom R, Eftekhari G, Rahimi-Balaei M, Khori V, Hajizadeh S, Dehpour AR, et al. (2013) The Role of α7 Nicotinic Acetylcholine Receptor in Modulation of Heart Rate Dynamics in Endotoxemic Rats. PLoS ONE 8(12): e82251. doi:10.1371/journal.pone.0082251
