# Supplementary material for: The Role of α7 Nicotinic Acetylcholine Receptor in Modulation of Heart Rate Dynamics in Endotoxemic Rats
Source: PLoS One. 2013 Dec 10;8(12):e82251. doi: 10.1371/journal.pone.0082251 (PMC3858293; doi:10.1371/journal.pone.0082251)
Supplement: File S1 — Contains: Appendix 1: The effect of endotoxin of expression of α7nACHR in rat atria and H9c2 cells. Appendix 2: The effect of pharmacological α7nACHR blockade on atrial expression of IL-1 and MCP-1 in rat atria. (DOCX) [file pone.0082251.s001.docx]

**Appendix 1: The effect of endotoxin of expression of α7nACHR in rat atria and H9c2 cells.**

We tested if there is any alteration in the expression of α7nACHR in rat atria or H9c2 cells upon LPS treatment.

For mRNA analysis H9c2 cells were incubated with either saline or LPS (10 ng/ml) for 24 h. mRNA was then extracted and cDNA was synthesized as described in method section. Expression of α7nACHR was assessed using RT-PCR. cDNA from rat atria was used as positive control. This experiment was repeated for at least three times. The results showed that incubation of H9c2 cells with endotoxin was unable to induce α7nAChR expression in this cell line (Figure S1A).

In another study, rats were given LPS (0.1 mg/kg or 1 mg/kg) or saline. The atria were isolated in oxygenated cold physiological salt solution 3 h post LPS injection. RNA was extracted and cDNA was synthesized as described in method section (5-8 rats were used in each group). The expression of α7nACHR was assessed using quantitative real time PCR. 18s ribosomal RNA was used as internal standard. We used different pairs of primers for α7nACHR (199 bp) that are suitable for real time PCR analysis. The sequences of primers are presented as follow: Rat α7nACHR, forward: 5’-atctgggcattgccagtatc-3’, reverse: 5’- tcccatgagatcccatcctc-3’. Rat 18s (as housekeeping gene), forward: 5’-atcaactttcgatggtagtcg-3’, reverse: 5’-tccttggatgtggtagccg-3’.

The results showed that α7nAChR mRNA level did not change upon LPS treatment in rat atria (Figure S1B)

**Figure S1.** A. The effect of incubation with LPS on expression of α7nACHR in H9c2 cells. cDNA from rat atrium was used as positive control. B. The effect of two doses of LPS on expression of α7nACHR in rat atria (5-8 atria were used in each group).

**Appendix 2: The effect of pharmacological α7nACHR blockade on atrial expression of IL-1 and MCP-1 in rat atria.**

In order to test the hypothesis that systemic α7nACHR blockade might affect expression of pro-inflammatory mediator in the atrium, we compared atrial expression of interleukin-1 (IL-1) and monocyte chemoattractant protein-1 (MCP-1) in control and endotoxemic rats given either saline or MLA. LPS (0.1 mg/kg, IP) was injected 30 min after IP administration of either MLA (5 mg/kg) or saline. The atria were isolated in oxygenated cold physiological salt solution 3 h post LPS injection. RNA was extracted and cDNA was synthesized as described in method section. Atria from saline treated rats were used as control. 5-8 rats were used in each group. The expression of IL-1 and MCP-1 was assessed using quantitative real time PCR. 18s ribosomal RNA was used as internal standard. The sequences of primers are presented as follow: Rat IL-1, forward: 5’-ggacccaagcaccttctttt-3’, reverse: 5’-agacagcacgaggcattttt-3’. Rat MCP-1, forward: 5’-gggcctgttgttcacagttgc-3’, reverse: 5’-gggacacctgctgctggtgat-3’. Rat 18s (as housekeeping gene), forward: 5’-atcaactttcgatggtagtcg-3’, reverse: 5’-tccttggatgtggtagccg-3’.

Our results showed that LPS (0.1 mg/kg) significantly increased both IL-1 and MCP-1 mRNA levels in rat atria (Figure S2). As shown in the Figure, pre-treatment with MLA was unable to significantly change atrial levels of these pro-inflammatory mediators.

**Figure S2.** The effect of pre-treatment with MLA on atrial IL-1 (A) and MCP-1 (B) mRNA levels in control and endotoxin-treated (0.1 mg/kg) rats. * P<0.05, ** P<0.01, *** P<0.001 in comparison with control groups (5-8 atria were used in each group).
